# Iopanoic acid rapidly restores euthyroidism in refractory thyrotoxicosis pre-thyroidectomy: a retrospective study

**DOI:** 10.1530/ETJ-25-0350

**Published:** 2026-03-18

**Authors:** Christine Newman, Muhammed Saqlain, Isra Ahmed Mohammed, Daniel Bell, Greta Lyons, Piyush Jani, Brian Fish, Diana Wood, Denise Tapa, David Halsall, Susan Oddy, Krishna Chatterjee, Nadia Schoenmakers, Carla Moran

**Affiliations:** ^1^ Wolfson Diabetes and Endocrine Centre, Cambridge University Hospitals NHS Foundation Trust, Cambridge, UK; ^2^ Pharmacy Department, Cambridge University Hospitals NHS Foundation Trust, Cambridge, UK; ^3^ Wellcome Trust-MRC Institute of Metabolic Science, University of Cambridge, Cambridge, UK; ^4^ Department of Ear Nose and Throat Surgery, Cambridge University Hospitals NHS Foundation Trust, Cambridge, UK; ^5^ Department of Clinical Biochemistry, Cambridge University Hospitals NHS Foundation Trust, Cambridge, UK

**Keywords:** iopanoic acid, thyrotoxicosis, thyroidectomy, Graves’ disease, amiodarone

## Abstract

**Objective:**

Restoration of euthyroidism in patients with thyrotoxicosis prior to thyroidectomy is recommended to decrease perioperative morbidity and thyroid storm risk. This is challenging when conventional anti-thyroid medication is contraindicated or ineffective. Iopanoic acid (IOPA), an iodinated contrast medium formerly used as an oral cholecystographic agent, may facilitate treatment of thyrotoxicosis, but limited manufacture precludes its use in the UK. We investigated the effectiveness and safety of IOPA for optimizing thyroid status in treatment-resistant thyrotoxicosis prior to thyroidectomy at a referral centre.

**Methods:**

With prior permission of our Drug and Therapeutics Committee, we sourced laboratory-grade (>98% pure) IOPA and administered this orally to control ongoing, severe thyrotoxicosis prior to thyroidectomy in 12 patients with inadequate response to standard therapies. Underlying aetiologies included Graves’ disease, amiodarone-induced thyrotoxicosis, toxic multinodular goitre and resistance to thyroid hormone beta. Medical case notes were reviewed retrospectively to analyse clinical and biochemical outcomes.

**Results:**

All patients exhibited a decline and normalization/near-normalization of free T3 (FT3) levels (mean 55% decrease, SEM 4.5%), with minimal changes in FT4 levels. Eleven patients proceeded to uneventful thyroidectomy, and IOPA was well tolerated with no directly attributable side effects.

**Conclusion:**

IOPA is a safe and effective agent for controlling biochemical thyrotoxicosis in preparation for thyroidectomy, including disease refractory to conventional medication. Highly pure, laboratory-grade iopanoic acid that is approved for human use is now available in the UK; consideration should be given to more widespread use for emergency treatment of life-threatening hyperthyroidism.

## Introduction

Thyrotoxicosis is a common endocrine disorder with diverse underlying pathologies, including Graves’ disease (GD), autonomous hormone synthesis (due to toxic adenoma or multinodular goitre, MNG), and thyroiditis (1). In addition, up to 12% of patients taking the anti-arrhythmic agent amiodarone develop amiodarone-induced thyrotoxicosis (AIT), either as a consequence of the high iodine content of amiodarone in pre-existing GD or MNG (type 1 AIT) or following a drug-induced destructive thyroiditis (type 2 AIT). Both AIT subtypes frequently coexist (2).

Thionamides (methimazole/carbimazole and propylthiouracil) are first-line therapy for GD and MNG, inhibiting thyroid hormone synthesis via thyroid peroxidase-mediated iodination, and are generally well tolerated. However, restoration of euthyroidism often takes weeks to months, making such therapy inadequate in life-threatening thyrotoxicosis where rapid control is required. Thionamides are not universally effective, even in GD, and treatment resistance is particularly common in type 1 and mixed subtypes of AIT. This contributes to high mortality rates in this condition, because affected patients invariably have pre-existing cardiac disease (1, 2, 3, 4). Serious adverse effects, including liver failure and agranulocytosis, may occur and contraindicate continued use.

Tissue-specific thyrotoxicosis in resistance to thyroid hormone beta (RTHβ) causes cardiac hyperthyroidism via intact TRα, with resultant risks of arrhythmias and cardiomyopathy (5, 6); in exceptional cases, thyroidectomy may be required.

Saturated iodine solutions are traditionally used whenever rapid restoration of euthyroidism is required, usually in preparation for definitive treatment with thyroidectomy; however, there is a limited repertoire of options when these fail.

Oral iodinated cholecystographic agents (OCAs), most commonly iopanoic acid (IOPA), were historically used to image the gallbladder before being replaced with more sensitive agents. Serendipitously, these agents can also ameliorate thyrotoxicosis, predominantly via the inhibition of type 1 deiodinase, which converts T4 to T3 in extrathyroidal tissue (7). IOPA has previously been used successfully in the short-term treatment of thyrotoxicosis but is no longer widely manufactured, precluding its routine use. However, laboratory-grade IOPA fit for human consumption is now available in the UK.

In this case series, we describe our experience of using IOPA treatment in patients with life-threatening thyrotoxicosis due to diverse underlying aetiologies, who required rapid restoration of euthyroidism prior to urgent thyroidectomy. All cases were treated with IOPA in a large tertiary referral centre in the UK, and biochemical response, safety, and efficacy were reviewed.

## Materials and methods

With prior permission of our Drug and Therapeutics Committee, we sourced laboratory-grade (>98% pure) IOPA powder and administered this pre-operatively with informed consent to control hyperthyroidism in 12 patients with treatment-resistant thyrotoxicosis between 2014 and 2023 at Cambridge University Hospital (CUH NHS Foundation Trust). We retrospectively reviewed the notes, and clinical and biochemical data were retrieved and analysed. This project was registered as a Clinical Audit with the Audit Department at CUH.

### Biochemical assays

All biochemical measurements were performed as part of routine clinical care and analysed using locally available assays. Thyroid function tests were analysed using Siemens Centaur XP assay (P1–P11) and Abbott Alinity (P12).

### Definitions

We defined ‘treatment-resistant thyrotoxicosis’ as thyrotoxicosis that persisted after treatment with appropriate doses of anti-thyroid drug(s), steroids (if the underlying aetiology was AIT), and iodine (if clinically indicated).

### Treatment

IOPA was first administered as a test dose of 50 mg (orally) to assess for acute adverse reactions related to its laboratory-grade status. Patients were then given 0.5 g doses of IOPA powder suspended in water, followed by fatty food to assist absorption. An initial dose of 0.5 g bd was increased to up to a maximum of 1 g tds if needed. In RTHβ, a starting dose of 0.5 g tds was used in anticipation of rising thyroid- stimulating hormone (TSH) levels due to hypothalamic–pituitary–thyroid axis resistance. Thionamides, steroids, and aqueous iodine oral solution (5% iodine and 10% potassium iodide, representing a total iodine content of 126 mg/mL) were continued, unless contraindicated. Patients were monitored for reported side effects, in particular thrombocytopaenia, hepatic and renal dysfunction, and gastrointestinal disturbance.

## Results

### Cases

Twelve adult patients (nine males; three females) were included; the median age was 45 years (range: 21–64); aetiologies included AIT (*n* = 5), Graves’ disease (*n* = 5), TNMG (*n* = 1), and RTHβ (*n* = 1). Patient demographics, biochemistry, and medication doses are summarized in Table 1, and case histories are summarized below. Reference ranges for thyroid biochemistry are as follows, unless otherwise stated – TSH: 0.35–5.5 mU/L (P1–P11) or 0.35–4.94 mU/L (P12), FT4: 10.5–21 pmol/L (P1, P4, P5, P8, P9, P10, and P11) or 10–19.8 pmol/L (P2, P3, P6, and P7) or 7.5–21.1 (P12), and FT3: 3.5–6.5 pmol/L (P1–P11) or 2.4–6.0 pmol/L (P12).

**Table 1:** Baseline and follow-up thyroid biochemistry, including response to iopanoic acid (IOPA) in all 12 cases. P5 took cholestyramine 4 g qds for 1 day and carbimazole 20 mg bd for 6 days prior to commencement of IOPA. The dose of aqueous iodine oral solution (5% iodine and 10% potassium iodide; total iodine content 126 mg/mL) was 0.3 mL three times daily, except where indicated. Case 3 died from a hospital-acquired pneumonia prior to surgery. Thrombocytopaenia and renal and liver dysfunction were felt to be due to causes unrelated to IOPA use in all affected cases.

Parameters	Cases											
	P1	P2	P3	P4	P5	P6	P7	P8	P9	P10	P11	P12
Sex	M	M	M	F	M	M	M	F	M	M	F	M
Age	50–60	60–70	60–70	20–30	50–60	30–40	50–60	50–60	30–40	30–40	40–50	20–30
Pathology	MNG	AIT	AIT	GD	GD	AIT	AIT	GD	AIT	RTHβ	GD	GD
Baseline thyroid biochemistry												
TSH (mU/L)	<0.03	<0.03	<0.03	<0.03	<0.03	<0.03	<0.03	<0.03	<0.03	2.5	<0.03	<0.1
FT4 (pmol/L)	51.9	50.1	51.6	75	33.3	100.5	79	52.1	64.9	42.6	40.6	52.3
FT3 (pmol/L)	7.6	NA	NA	>30.8	16.6	14.2	17.9	>30	13.6	17.5	>30.8	>30.7
Maximal ATM daily dose before commencing IOPA												
PTU (mg)	600	-	900	-	-	900	1,200	-	-	-	-	- (*)
CBZ (mg)	-	60	-	- (*)	40	-	-	70	40	30	60	-
Dexamethasone (mg)	1	3	4	-	-	^†^	3	-	2	-	-	-
Duration (days) of AIOS before commencing IOPA	5	5	5	13	19	7	8	5	-	8	10	0.2 mL tds (8 days)
							0.36 g KI (2 days)					0.3 mL tds (5 days)
Thyroid biochemistry before commencing IOPA												
TSH (mU/L)	<0.03	<0.03	<0.03	<0.03	<0.03	<0.03	<0.03	<0.03	<0.03	9.2	<0.03	<0.03
FT4 (pmol/L)	59.4	56.8	84.1	37.9	54.6	85	60.4	29.7	132.9	36.1	16.9	42.9
FT3 (pmol/L)	12.8	8.8	6.7	13.4	30.8	21.8	7.5	14	14.6	16.5	7.7	13.5
IOPA duration and daily dose pre-op												
Duration (days)	13	8	10	2	4	11	4	2	4	3	0.5	7
Total daily dose (g)	1	1 (2 days)	1	1	1	1.5	1 (1 day)	1	1	1.5	0.5	1 (2 days)
		1.5 (6 days)					1.5 (3 days)					1.5 (2 days)
												3 (3 days)
Thyroid biochemistry before surgery												
TSH (mU/L)	<0.03	<0.03	<0.03	<0.03	<0.03	<0.03	<0.03	<0.03	<0.03	14.53	NA	<0.03
FT4 (pmol/L)	85.4	65.5	115.3	32.7	49.8	67.3	38.8	27.9	129.7	43.7	NA	55.9
FT3 (pmol/L)	5	4.8	4.9	6.0	7	5.0	3.3	6.3	5.6	7.5	NA	8.4
Screening for reported adverse effects of IOPA												
Platelets	N	Low	Low	N	N	N	N	N	N	Low	N	N
Renal function	N	N	CRF	N	N	N	N	N	AKI	N	N	N
Liver dysfunction	No	Yes	NA	No	No	Yes	Yes	NA	Yes	No	Yes	Yes

ATM, anti-thyroid medication; IOPA, iopanoic acid; AIOS, aqueous iodine oral solution; MNG; multinodular goitre, AIT, amiodarone-induced thyrotoxicosis; GD, Graves’ disease; RTHβ, resistance to thyroid hormone beta; KI, potassium iodide; CRF, chronic renal failure; AKI, acute kidney injury; N, normal result (within reference interval); and NA, not available.

* Indicates neutropaenia due to anti-thyroid drugs.

^†^ Indicates prednisolone use, 30 mg daily.

Reference ranges – TSH: 0.35–5.5 mU/L, FT4: 10.5–21 pmol/L (P1, P4, P5, P8, P9, P10, P11, and P12) or 10–19.8 pmol/L (P2, P3, P6, and P7), FT3 3.5–6.5 pmol/L. P12 baseline RR – TSH: 0.35–4.94 mU/L, FT4: 7.5–21.1 pmol/L, and FT3: 2.4–6.0 pmol/L.

### Case histories

**P1:** an individual with massive multi-nodular goitre was admitted with fast atrial fibrillation (rate 180 bpm) and dilated cardiomyopathy. Compliance with propylthiouracil (PTU) had been poor after cessation of carbimazole due to drug intolerance. Biochemical thyrotoxicosis (Table 1) progressed despite PTU 200 mg tds (peak FT4: 113.9, FT3: 17.5 pmol/L, and TSH: < 0.03 mU/L). Low-grade ^99m^Tc uptake (total 0.7%) suggested thyroiditis; however, response to dexamethasone and aqueous iodine oral solution (0.3 mls tds for 5 days) were poor (Table 1). Treatment with IOPA normalized FT3 within 24 h (TSH: < 0.03 mU/L, FT4: 57.5 pmol/L, and FT3: 5.6 pmol/L), and IOPA was continued alongside PTU and dexamethasone for 13 days prior to successful thyroidectomy.

**P2:** an adult male developed AIT, with low vascularity on ultrasound, on a background of dilated cardiomyopathy and atrial fibrillation. He responded poorly to carbimazole (20 mg thrice daily), dexamethasone, and five days of aqueous iodine oral solution (0.3 mls tds) (Table 1). IOPA normalized FT3 (FT3: 5.5, FT4: 68 pmol/L) after 24 h (three doses); the medication was continued for 8 days prior to successful thyroidectomy.

**P3:** an adult male developed AIT in the setting of pre-existing dilated cardiomyopathy and failed to respond to carbimazole and dexamethasone, resulting in hospital admission with thyrotoxic myopathy (FT4: 76.4, FT3: 7.5 pmol/L). Substitution of carbimazole for PTU and an increase in dexamethasone dose failed to restore euthyroidism (FT4: 76.1, FT3: 7.0 pmol/L), and despite five days of aqueous iodine oral solution (0.3 mls tds), his biochemistry worsened (FT4: 96.4, FT3: 8.8 pmol/L). IOPA achieved sustained normalization of FT3 within 24 h (FT4: 88.9, FT3: 4.6 pmol/L), but he then became acutely unwell, precluding surgical intervention and died of a hospital-acquired pneumonia.

**P4:** cessation of carbimazole due to thionamide-induced neutropaenia resulted in recurrent thyrotoxicosis in an adult female with Graves’ disease. She was commenced on aqueous iodine oral solution (0.3 mls tds), in preparation for total thyroidectomy with commencement of IOPA after 13 days due to inadequate response. Within two days, her FT3 normalized and she underwent uncomplicated surgery.

**P5:** an adult male with recurrent Graves’ disease developed refractory thyrotoxicosis following non-specific intolerance of carbimazole and PTU-induced rash (Table 1). Despite aqueous iodine oral solution (taken with unconfirmed adherence for two weeks, 0.3 mls tds), he remained thyrotoxic (FT4: 23.2, FT3: 8.3 pmol/L), precluding thyroidectomy. He then developed rebound thyrotoxicosis, despite addition of cholestyramine 4 g qds for 24 h and carbimazole 20 mg bd for seven days and was hospitalized with fast atrial fibrillation (FT4: 54.6, FT3: > 30 pmol/L). IOPA near-normalized FT3 within four days, permitting uneventful thyroidectomy (FT4: 49.8, FT3: 7.0 pmol/L).

**P6:** an adult male with previous cardiac transplant due to congenital heart disease developed AIT (Table 1). He required rapid treatment and was referred for emergency thyroidectomy. Eleven days treatment with IOPA 0.5 g tds and anti-thyroid drugs achieved a sustained normalization of FT3 within eight days (FT4 83.7, FT3 5.8 pmol/L), permitting successful thyroidectomy.

**P7:** an individual with transposition of the great vessels and severe systolic dysfunction had been commenced on amiodarone two years previously following a ventricular fibrillation (VF) arrest and implantable cardioverter defibrillator (ICD) placement. He developed AIT with inadequate response to escalating doses of PTU and dexamethasone over a two-month period. Addition of aqueous iodine oral solution, 1.2 mls daily, for eight days, and additional 360 mg potassium iodide, for two days, achieved minimal improvement (FT4: 60.4, FT3: 7.5 pmol/L), and a rising alanine transaminase (481 U/L, NR: 7–40) necessitated cessation of PTU. IOPA normalized FT3 within 24 h (FT4: 49.6, FT3: 4.3 pmol/L, and TSH: < 0.03 mU/L), and euthyroidism was maintained with 0.5 g IOPA tds for three days prior to uneventful thyroidectomy. Hepatic dysfunction subsequently recovered.

**P8:** an individual with refractory Graves’ disease, following ten weeks of carbimazole therapy, was admitted with worsening tachycardia. Limited improvement after six days inpatient carbimazole treatment prompted commencement of aqueous iodine oral solution (0.3 mls tds), which achieved a FT3 plateau of 14.0 pmol/L after 5 days. Addition of IOPA normalized FT3 within 48 h with subsequent uneventful thyroidectomy.

**P9:** an individual with cardiomyopathy required ICD insertion and commencement of amiodarone for ventricular tachycardia. He later developed AIT with cardiac decompensation, which initially responded to carbimazole and dexamethasone (FT4: 38, FT3: 6.4 pmol/L). Eleven days later, however, he developed refractory thyrotoxicosis with worsening cardiac instability and oedema (FT4: 98.4, FT3: 14.1 pmol/L), despite increasing dexamethasone dose (FT4: 129.2, FT3: 17.0 pmol/L). IOPA 0.5 g bd normalized FT3 within 24 h (FT4: 137.7, FT3: 6.2 pmol/L) and maintained normal FT3 levels until surgery four days later (FT4: 129.7, FT3: 5.6 pmol/L).

**P10:** an individual with elevated thyroid hormones due to RTHβ (heterozygous mutation in *THRB*, L454FfsTer11) developed dilated cardiomyopathy, felt to be due to relative cardiac thyrotoxicosis. He received eight days of aqueous iodine oral solution (0.3 mls tds) in preparation for thyroidectomy, but rising TSH levels limited its effectiveness. IOPA 0.5 g tds achieved a nadir FT3 level of 6.6, FT4 level of 41 pmol/L, and TSH level of 15.92 mU/L after 48 h, rising to FT4 43.7, FT3 7.5 pmol/L, and TSH 14.53 mU/L the following day, prior to successful thyroidectomy.

**P11:** an individual developed Graves’ disease and moderate ophthalmopathy, which was refractory to carbimazole treatment. Aqueous iodine oral solution (0.3 mls tds) achieved a nadir FT3 of 7.5 pmol/L after 6 days (FT4: 19.1 pmol/L; TSH: < 0.03 mU/L), which rose to 7.7 pmol/L on the 7th day (FT4: 16.9 pmol/L; TSH: < 0.03 mU/L). A single dose of 0.5 g IOPA was given to block peripheral conversion of FT4 to FT3 prior to thyroidectomy the following morning without repeat thyroid function tests. Surgery was complicated by hypoparathyroidism but was otherwise uneventful.

**P12:** an individual developed Graves’ disease on a background of previous thrombolysis for embolic stroke due to infective endocarditis, with a subsequent intracranial bleed resulting in paraplegia, tracheostomy, and nasogastric feeding. Mitral valve repair was delayed due to thyrotoxicosis (FT4: 52.3, FT3: > 30 pmol/L, and TSH: < 0.01 mU/L), and thyroidectomy was scheduled after development of agranulocytosis on PTU. He initially responded to aqueous iodine oral solution (0.2 mL tds), but this response plateaued on days 9 and 10 (day 9 – FT4: 39.1, FT3: 13.5 pmol/L, and TSH: < 0.01 mU/L; day 10 – FT4: 42.9 pmol/L, RR: 10.5–21, FT3: 13.5, RR: 3.5–6.5 pmol/L, and TSH: < 0.03 mU/L, RR: 0.35–5.5) despite an increase in aqueous iodine oral solution to 0.3 mL tds on day 8. IOPA, administered via a nasogastric tube, decreased FT3 levels within 24 h (FT3: 7.5 pmol/L, RR: 3.5–6.5; FT4: 39 pmol/L, RR: 10.5–21) but FT3 subsequently rose over the next 2 days (FT3: 10.3 pmol/L, RR: 3.5–6.5; FT4: 42.4 pmol/L, RR: 10.5–21). Aqueous iodine oral solution was stopped after a total course of 13 days, and an increase in the dose of IOPA to 0.5 g tds resulted in a transient FT3 decrease after 24 h (FT3: 7.8 pmol/L, RR: 3.5–6.5; FT4: 44.7 pmol/L, RR: 10.5–21) followed by increasing levels the next day (FT3: 10.1 pmol/L, RR: 10.5–21; FT4: 52.4 pmol/L, RR: 10.5–21; and TSH: < 0.03 mU/L, RR: 0.35–5.5). The dose of IOPA was increased further to 1 g tds, and following further fluctuations in thyroid function, he proceeded to thyroidectomy three days later with FT3 = 8.4 pmol/L, RR: 3.5–6.5; FT4 = 55.9 pmol/L, RR: 10.5–21; and TSH: < 0.03 mU/L, RR: 0.35–5.5.

**Figure 1:**
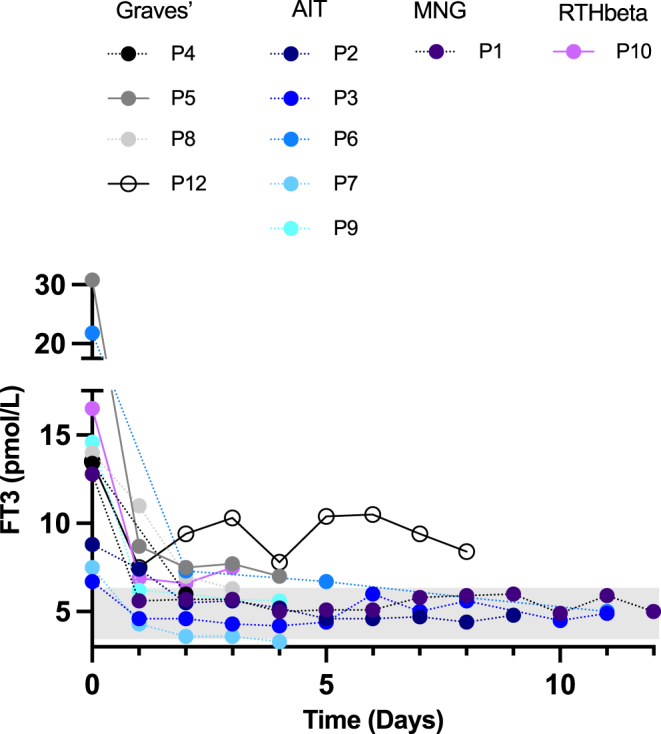
Daily FT3 levels immediately before and following commencement of IOPA until surgery. Patients are subdivided according to the aetiology of their thyrotoxicosis. P11 did not have thyroid biochemistry measured while on iopanoic acid.

The mean FT3 level was 14 pmol/L prior to IOPA (range: 6.7–30.8 pmol/L, RR: 3.5–6.5) and decreased in all patients after IOPA administration (mean decrease = 55%, range: 26–77%, measured in 11/12 patients) (Fig. 1). FT3 level normalized (8/11) or near-normalized (3/11) on IOPA after an average of 2.1 days (range: 1–8) (Fig. 1). The mean duration of IOPA use was 5.8 days (range: 0.5–12). Cases in whom FT3 did not normalize included P5, who had rebound thyrotoxicosis following escape from the effects of aqueous iodine oral solution; P10, who had an increase in TSH following commencement of IOPA in the context of RTHβ; and P12, who may have been escaping from the Wolff–Chaikoff effect following a prolonged course of aqueous iodine oral solution. In these individuals, following commencement of IOPA, FT3 had either plateaued (P5 and P10) or the dose of IOPA was increased due to a rising FT3 level on lower IOPA doses.

**Figure 2:**
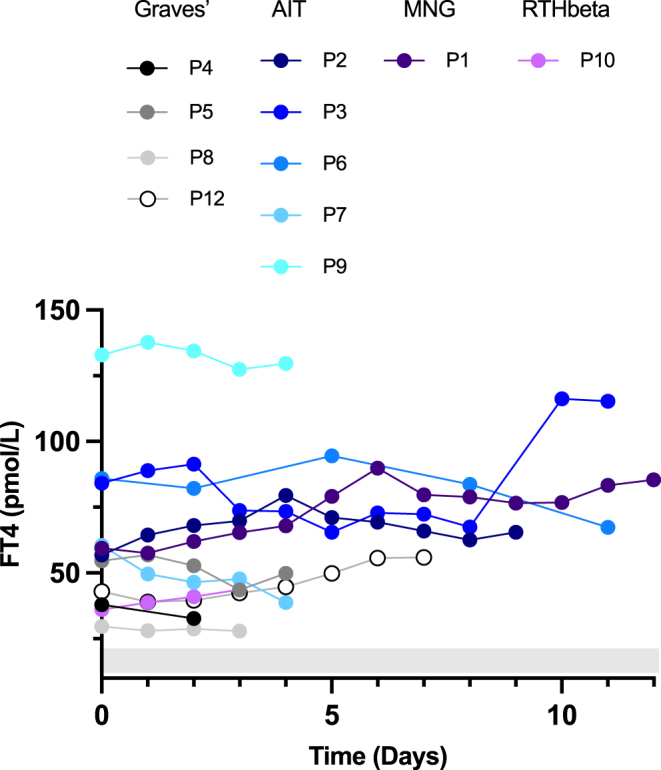
Daily FT4 levels immediately before and following commencement of IOPA until surgery. Patients are subdivided according to the aetiology of their thyrotoxicosis. P11 did not have thyroid biochemistry measured while on iopanoic acid.

The mean FT4 level prior to IOPA was 58.1 pmol/L (range: 16.9–132.9, RR: 10.5–21 for P1, P4, P5, P8, P9, P10, and P11; 10–19.8 for P2, P3, P6, and P7; and 7.5–21.1 for P12). Unlike FT3 levels, FT4 levels usually remained static (mean = 64.7 pmol/L post-IOPA, range: 27.9–129.7), with mean FT4 at the time of surgery being 105% of baseline (range: 64–144%), Fig. 2. In three cases with declining FT4 levels at the time of surgery, there was an earlier, transient increase in FT4 levels (percentage baseline FT4 P5: 104% on day 1, P6: 110% on day 5, and P9: 104% on day 1 of treatment).

IOPA was well tolerated with no gastrointestinal side effects. Liver function did not deteriorate in patients with (P3 and P7) or without pre-existing liver dysfunction, except in P9, in whom an acute deterioration in liver function improved prior to cessation of IOPA and was likely due to congestive hepatopathy. Renal function remained normal in all except one patient (P9), in whom high-dose furosemide for cardiac failure was the most likely cause of an acute kidney injury, which later reverted to normal post-operatively. Three patients (P2, P3, and P10) had mild thrombocytopaenia prior to starting IOPA (92–105 × 10^9^/L, range: 150–450), which did not worsen in response to IOPA and was likely related to coexistent pulmonary hypertension and thyrotoxicosis. A single pre-operative death was due to a hospital-acquired pneumonia.

## Discussion

Although primary hyperthyroidism is readily treatable in the majority of cases, severe thyrotoxicosis is an endocrine emergency, which may be associated with significant mortality, especially in patients with underlying cardiac disease. There is a limited repertoire of therapies for refractory disease or to replace conventional medications when these are contraindicated, and moreover, the time required for thionamides to achieve endocrine control may be excessive if there is systemic or cardiac decompensation (1). This is particularly relevant in AIT where effectiveness of thionamides may be limited by high intrathyroidal iodine content and patients often have significant cardiac morbidity (2). In rare forms of hyperthyroxinaemia (e.g. RTHβ), optimal management of cardiac thyrotoxicity is relatively uncharted (6).

The use of alternatives or adjuncts to thionamides is often limited by side effects or modest efficacy. Aqueous iodine rapidly blocks thyroid hormone synthesis via the Wolff–Chaikoff effect, but escape may trigger rebound hyperthyroidism (8). Perchlorate inhibits NIS-mediated iodide uptake but carries serious adverse effects, including aplastic anaemia, limiting long-term use (9). High-dose glucocorticoids modestly reduce T4 to T3 conversion, with associated significant side effects (9). Cholestyramine sequesters T4 in the gut, achieving a gradual, mild-to-moderate decrease in FT4 levels (up to 13% when used in combination with methimazole for 4 weeks) (10, 11). Lithium inhibits thyroid hormone release and intrathyroidal iodine turnover, normalizing FT4/FT3 in 2–3 weeks, and may enhance radioiodine sensitivity, but has an extensive side effect profile (2, 11, 12). Plasma exchange rapidly removes thyroid hormones but is invasive, requiring specialized resources (13). There remains a clear need for rapidly acting, effective anti-thyroid therapies with minimal side effects.

OCAs, including IOPA and sodium ipodate, have been used previously for rapid correction of thyrotoxicosis in Graves’ disease (neonates and adults) (14, 15), AIT (3, 4, 16), exogenous hyperthyroidism (17), subacute thyroiditis (18), thyrotropinoma (19), and thyrotoxicosis of pregnancy (20). In many of these settings, the OCA has been used to optimize thyroid status prior to thyroidectomy (3, 15, 16, 19, 20). IOPA is usually well tolerated, and although gastro-intestinal upset is common, severe adverse effects reported in patients receiving oral cystographic agents at high doses (3–6 g) for imaging (renal failure and thrombocytopaenia) are rare at the lower doses (1 g daily) used for thyrotoxicosis (15, 18, 21, 22).

We describe twelve patients successfully treated with IOPA pre-operatively, including patients with Graves’ disease or MNG in whom there were contraindications (agranulocytosis) or refractoriness to thionamides and patients with life-threatening AIT unresponsive to conventional treatment. Our cohort was unique in including one patient with rebound thyrotoxicosis following escape from the Wolff–Chaikoff effect on aqueous iodine oral solution and another patient with life-threatening cardiac thyrotoxicity due to RTHβ. In keeping with previous reports, IOPA was well tolerated. The daily cost of iopanoic acid at a dose of 500 mg bd was approximately £120 (Cambridge Bioscience, UK).

In previous reports, IOPA has striking effects on serum T3, achieving a decrease in serum FT3 from 36 to 77% within six to twelve hours and normalizing FT3 in two to five days (23). Here, IOPA used in combination with conventional medications was similarly effective in achieving a marked and rapid reduction in FT3 levels. This effect occurs due to competitive inhibition of peripheral type 1 deiodinase by IOPA, and the resulting blockade of FT4 conversion to FT3 occurs independently of any direct thyroidal effect (7). This may be particularly advantageous in situations requiring rapid restoration of euthyroidism or in settings (thyroiditis and exogenous hyperthyroidism) where modulating endogenous thyroid hormone synthesis is ineffective. In addition, in patients with poor adherence to therapy, treatment adherence may be less challenging with a short-term course of IOPA, which could be administered either with inpatient observation or with close biochemical monitoring on an outpatient basis (15, 17, 18, 20).

The effects of IOPA on the prohormone FT4 are more complex. The hepatic metabolism of IOPA liberates inorganic iodine (650 mg iodine per gram of IOPA), which may decrease thyroidal synthesis and release of thyroid hormones through the Wolff–Chaikoff effect in addition to decreasing thyroid vascularity (7, 8, 15). Inhibition of T4 secretion will tend to decrease serum T4 levels; however, conversely, IOPA may also impair hepatic uptake of T4 and displace it from hepatic binding sites, tending to increase serum T4 levels (24). Consistent with other reports, and perhaps due both to hepatic and thyroidal actions, FT4 levels declined more modestly than FT3 levels in our patients and a transient, early rise in FT4 levels was sometimes observed on IOPA (3, 20, 25). However, even markedly elevated FT4 levels (e.g. in P9) did not compromise surgical outcomes due to the highly effective blockade of FT4 to FT3 conversion.

IOPA may have been less effective in P5 and P12 due to their prolonged courses of aqueous iodine oral solution (19 and 13 days, respectively) with rebound thyrotoxicosis in P5 and a plateauing FT3 twice the upper limit of normal in P12, suggesting impending escape from the Wolff–Chaikoff effect at the time IOPA was started. In P12, IOPA administration via a fine bore nasogastric tube may also not have been as effective as oral administration. Escape from the Wolf–Chaikoff effect usually occurs 7–10 days after aqueous iodine oral solution is commenced and is mediated by a decrease in sodium/iodide symporter expression, with the concomitant decrease in intrathyroidal iodide levels permitting enhanced thyroid hormone synthesis from residual iodine substrate (8). It is likely that such an accelerated TH synthesis may eventually exceed the blockade of IOPA on T4-to-T3 conversion and any thyroidal inhibitory effect of the iodine content of IOPA itself may be abrogated. These observations suggest that in patients taking aqueous iodine oral solution, IOPA should be employed early in the treatment regimen prior to surgery.

In RTHβ, cardiovascular complications may shorten life expectancy, with isolated cases of life-threatening cardiomyopathy (5, 6). Lowering TH may benefit selected patients, but thionamides cause rebound TSH elevation and goitrogenesis (6, 26). Thyroidectomy is generally avoided due to later difficulty maintaining euthyroidism, but in this case, elevated thyroid hormones caused life-threatening cardiomyopathy. IOPA substantially reduced FT3 and was more effective than aqueous iodine, although FT3 remained elevated, likely due to compensatory TSH stimulation of hormone synthesis.

IOPA has been used successfully both in isolation and as an adjunct to conventional treatment in diverse forms of thyrotoxicosis (3, 4, 18, 19). However, it has been suggested that, in AIT type 2, euthyroidism may be achieved more rapidly with glucocorticoids than with IOPA (16). Intriguingly, although there is a strong theoretical concern that the high iodine load of IOPA may adversely affect response to subsequent radioiodine treatment in hyperthyroidism, a randomized control trial involving 200 patients suggested that this may not be the case (27).

IOPA is generally considered a short-term option for thyrotoxicosis due to the high rate of escape during long-term use. Although some patients have remained euthyroid for nearly 2 years, two studies using 0.5 g IOPA in Graves’ disease showed concerning outcomes. Wang *et al.* studied 40 patients: all improved initially, but FT3 rose in 22 after 1 month, and 18 developed marked hyperthyroidism within 6 months (28). In a smaller study of 12 patients, five remained euthyroid for 22 months, but seven exhibited rising T3/T4 after 14–42 days, and two responded poorly to later methimazole administration (29). FT3/FT4 ratio increases during escape suggest involvement of 5′-deiodinase blockade, while FT4 rises also indicate escape from iodine-mediated inhibition. These high relapse rates and subsequent refractoriness support using IOPA only as a short-term intervention, typically before definitive therapy.

Our study has several limitations: it is retrospective, involves a small cohort with diverse aetiologies of thyrotoxicosis, and was conducted at a single, high-volume tertiary centre, which may limit generalizability. Nevertheless, it offers valuable insights into the use of iopanoic acid as an adjunct in severe thyrotoxicosis refractory to standard therapies.

## Conclusion

Our study supports the use of IOPA as a highly effective and safe agent for controlling biochemical thyrotoxicosis refractory to other agents, as a short-term prelude to thyroid surgery. Highly pure, laboratory-grade iopanoic acid that is approved for human use is now available in the UK and could be encapsulated for hospital pharmacies to stock this agent for emergency use in uncontrolled, life-threatening hyperthyroidism.

## Declaration of interest

The authors have the following potential conflicts of interest to declare. NS has been a member of the scientific advisory board for Egetis Therapeutics. CM performs honorary consultancy work for Egetis Therapeutics, has received publication fees from Institut Biochimique SA (IBSA), and acts on an *ad hoc* basis as an expert advisor to the Health Products Regulatory Agency of Ireland.

## Funding

NS and KC are supported by the Wellcome Trusthttps://doi.org/10.13039/100010269 (Investigator Award 210755/Z/18/Z to KC; Senior Fellowship 219496/Z/19/Z to NS) and NIHR Cambridge Biomedical Research Centre.

## Author contribution statement

MS, IAM, DB, PJ, BF, DFW, DT, DH, SO, KC, NS, and CM were involved in clinical case management. CN, MS, NS, and IAM collated and analysed the data, and CN, NS, and CM wrote the manuscript. All authors read and approved the final version.

## Patient consent

Written informed consent for publication of clinical details has been obtained from patients who may potentially be identified from the case descriptions.

## Data access statement

The dataset reported in this article is not available for sharing. Participants did not provide informed consent for broader data sharing and further disclosure of the data could risk the identification of individual patients.

## Statement of ethics

This research was conducted ethically in accordance with the World Medical Association Declaration of Helsinki. No formal ethical approval was required, since all investigations and care provided was performed during the course of routine clinical care.
